# Supplementary material for: Industrial chicory genome gives insights into the molecular timetable of anther development and male sterility
Source: Front Plant Sci. 2023 Jun 13;14:1181529. doi: 10.3389/fpls.2023.1181529 (PMC10298185; doi:10.3389/fpls.2023.1181529)
Supplement: Supplementary file 1 [file DataSheet_1.pdf]

Supplemental material for:

Waegneer E, Rombauts S, Baert J, Dauchot N, De Keyser A, Eeckhaut T, Haegeman A, Liu C, Maudoux O, Notté C, Staelens A, Van der Veken J, Van Laere K and Ruttink T. (2023). Industrial chicory genome gives insights into the molecular timetable of anther development and male sterility. **Frontiers in Plant Science**.

Supplemental Table 1: Genome assembly and gene annotation statistics from the L8001_v1 genome.

Chromosome assembly statistics L8001_v1

Sequence ID	Sequence length (nt)	GC content (%)	Number of contigs
LG1	105.806.898	35.22	561
LG2	78.968.886	34.82	360
LG3	114.748.645	35.06	616
LG4	72.816.357	35.04	320
LG5	76.342.043	34.90	344
LG6	124.886.246	34.98	655
LG7	88.382.747	34.87	368
LG8	127.960.131	34.70	561
LG9	82.636.264	34.88	363
unanchored	49.072.383		

General genome statistics L8001_v1

genome size (scaffolds):	921.620.600	nt		
genome size (contigs):	919.456.645	nt (excluding N)		
largest scaffold:	127.960.131	nt		
average scaffold lenght:	239.568	nt		
number of contigs:	8.220			
largest contig:	2.303.520	nt		
average contig lenght:	111.856	nt		
gaps (>50N):			4322	(2.163.900 nt)
L50 - N50 :	4		106.331.623	
L75 - N75 :	7		79.387.611	
L90 - N90 :	9		73.242.076	

Gene annotation statistics L8001_v1

number of loci (exons+introns):	53.960	
average length loci:	2.502	
loci density:	17.307	nt/gene
number of genes:	53.386	
gene density:	58	genes/Mb
average length genes:	970	nt
median length genes:	720	nt
number of exons:	266.519	
GC content of CDS:	34,96	%
cumulative CDS length:	49.917.919	nt
average length CDS:	935	nt
cumulative exon length:	51.765.486	

average length exons:	194	nt
median length exons:	115	nt
longest exons:	12.694	nt (cicin03g25730.1.6)
average number of exons per gene:	4,99	
most exons per gene:	77	(cicin02g11390.1)
longest CDS:	14.370	nt
shortest CDS:	195	nt
cumulative intron length:	78.336.357	nt
average length intron:	369	nt
median length introns:	111	nt
longest intron:	24.895	nt (cicin07g03140.1.5-cicin07g03140.1.4)
GC content of intron:	35,08	%

Supplemental Table 2: Genome assembly statistics from the genome assembly from Fan et al. (2022). Sequences were extracted from NCBI (Bioproject ID PRJNA798105).

Chromosome assembly statistics

Sequence ID	L8001_v1 chromosome	Sequence length (nt)	GC content (%)	Number of contigs
CM042009.1	LG8	161.702.566	35.09	772219
CM042010.1	LG3	159.989.680	35.70	748472
CM042011.1	LG6	157.584.665	35.22	757852
CM042012.1	LG1	142.005.817	35.80	654754
CM042013.1	LG2	126.222.701	36.06	588371
CM042014.1	LG9	121.338.966	35.65	586290
CM042015.1	LG5	119.386.547	35.02	561236
CM042016.1	LG7	117.937.712	35.05	545064
CM042017.1	LG4	98.217.271	35.30	474603
unanchored		74.366.644		

General genome statistics

genome size (scaffolds):	1.278.752.569	nt
genome size (contigs):	665.877.180	nt (excluding N)
largest scaffold:	161.702.566	nt
average scaffold length:	6.147.849	nt
number of contigs:	5.961.809	
largest contig:	7.815	nt
average contig lenght:	111,69	nt
gaps (>50N):	93	(612.875.389 nt)

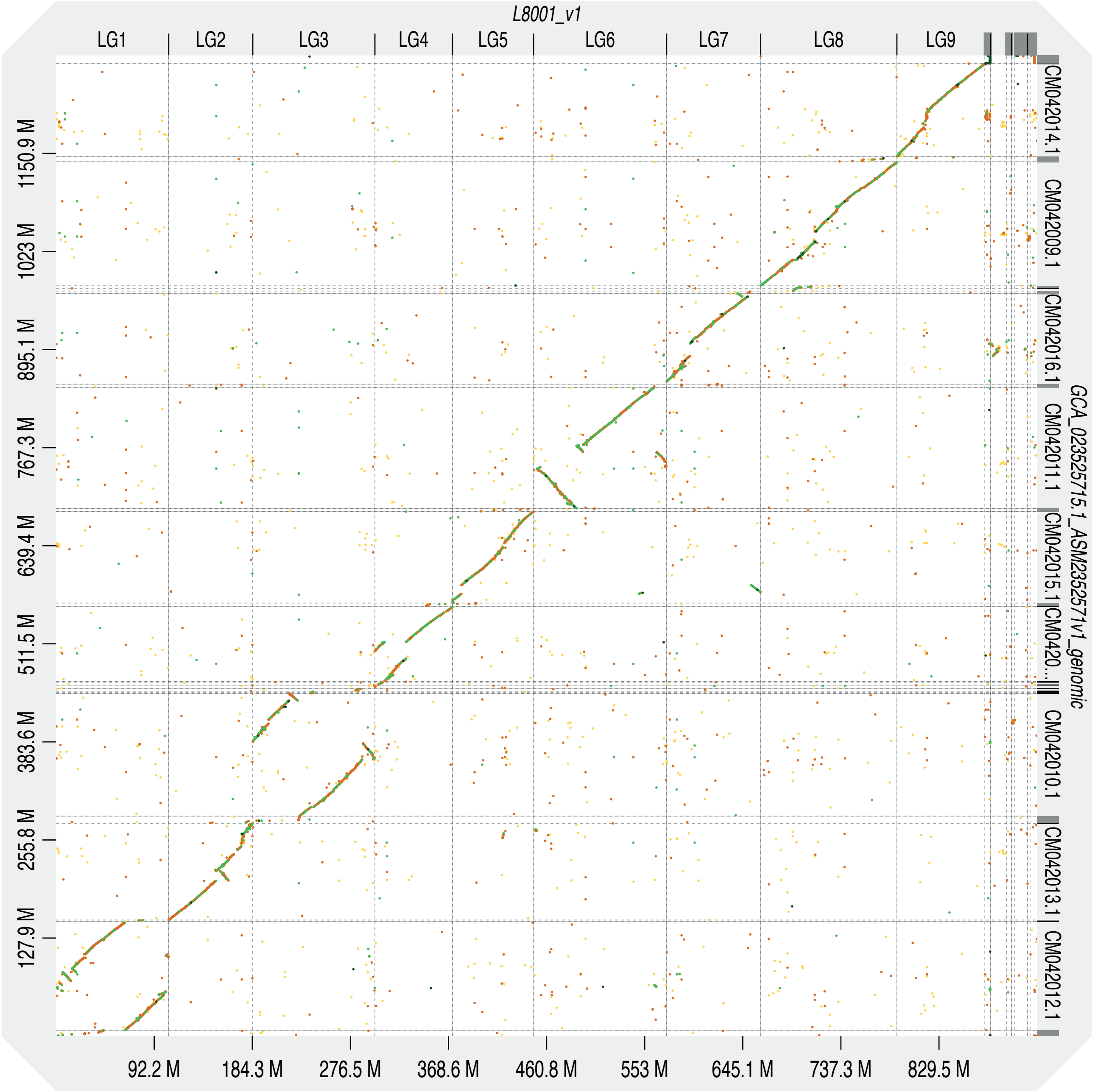

Supplemental Figure 1: Dotplot comparing L8001_v1 genome assembly (this study) to the genome assembly published by Fan et al., (2022). Dotplot was generated with D-genies, based on minimap2 alignments.

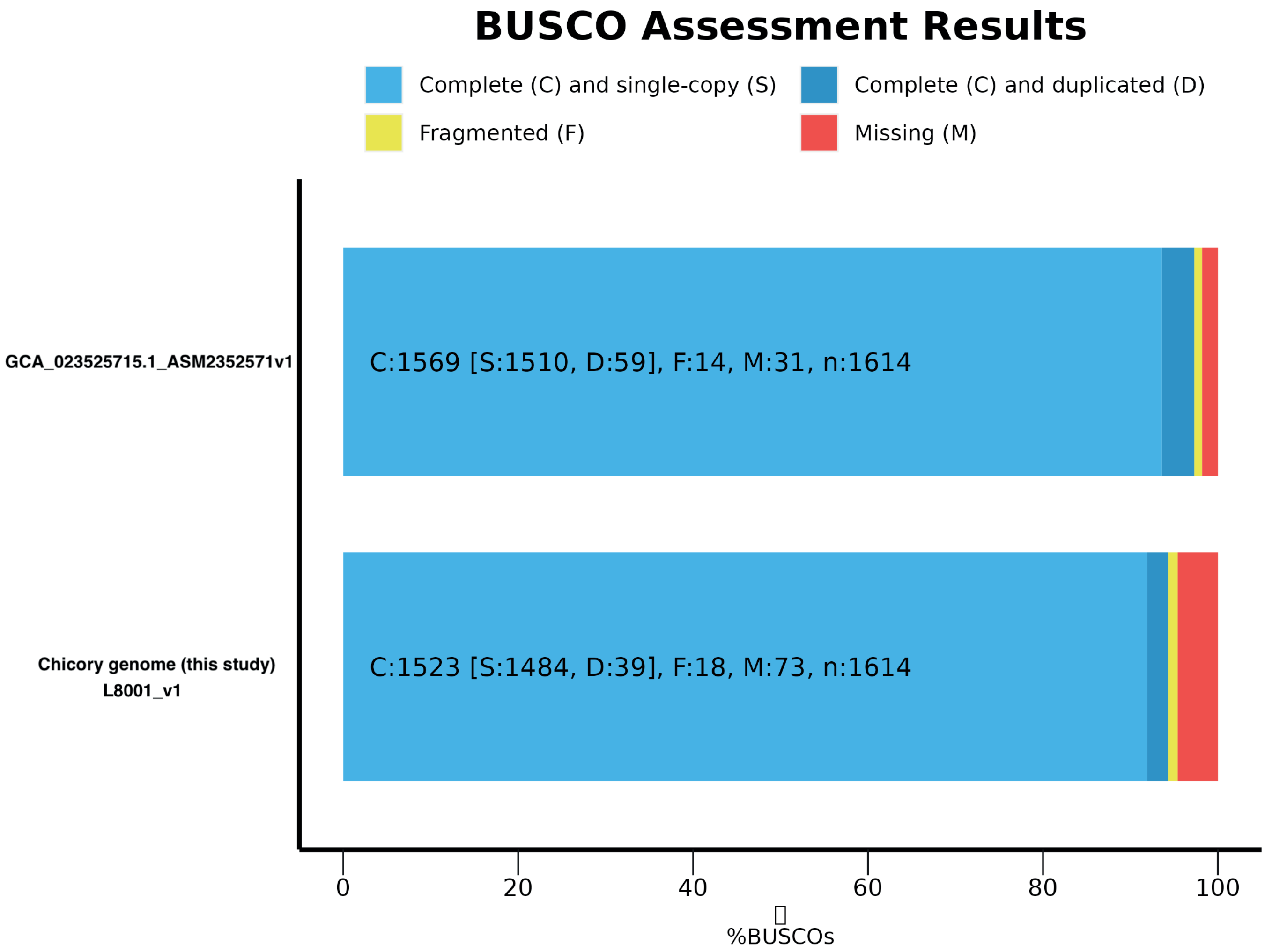

Supplemental Figure 2: Comparison of BUSCO analysis between L8001_v1 genome assembly (this study) to the genome assembly published by Fan et al., (2022).

Reference

FAN, W., WANG, S., WANG, H., WANG, A., JIANG, F., LIU, H., ZHAO, H., XU, D. & ZHANG, Y. 2022. The genomes of chicory, endive, great burdock and yacon provide insights into Asteraceae palaeo-polyploidization history and plant inulin production. Molecular Ecology Resources, 22, 3124-3140. doi: https://doi.org/10.1111/1755-0998.13675
